# Effects of high pressure on the electrical resistivity and dielectric properties of nanocrystalline SnO_**2**_

**DOI:** 10.1038/s41598-018-22965-8

**Published:** 2018-03-23

**Authors:** Wenshu Shen, Tianji Ou, Jia Wang, Tianru Qin, Guozhao Zhang, Xin Zhang, Yonghao Han, Yanzhang Ma, Chunxiao Gao

**Affiliations:** 1 State Key Laboratory for Superhard Materials, Jilin University, Changchun, 130012 China; 2 Key Laboratory of Functional Materials Physics and Chemistry of the Ministry of Education, Jilin Normal University, Siping, 136000 China; 3 Department of Mechanical Engineering, Texas Tech University, Lubbock, Texas 79409 USA

## Abstract

The electrical transport and structural properties of tin oxide nanoparticles under compression have been studied by *in situ* impedance measurements and synchrotron X-ray diffraction (XRD) up to 27.9 GPa. It was found that the conduction of SnO_2_ can be improved significantly with compression. Abnormal variations in resistivity, relaxation frequency, and relative permittivity were observed at approximately 12.3 and 25.0 GPa, which can be attributed to pressure-induced tetragonal- orthorhombic-cubic structural transitions. The dielectric properties of the SnO_2_ nanoparticles were found to be a function of pressure, and the dielectric response was dependent on frequency and pressure. The dielectric constant and loss tangent decreased with increasing frequency. Relaxation-type dielectric behaviour dominated at low frequencies. Whereas, modulus spectra indicated that charge carrier short-range motion dominated at high frequencies.

## Introduction

Transparent conductive oxides (TCOs) attract increasing attention in modern optoelectronic devices due to their large optical transparency and high electrical conductivity^1^. SnO_2_ is one of the widely used TCOs, with a bandgap E_g_ of ~3.6 eV. It shows high electrical conductivity and optical transparency in the visible region, making it useful in optoelectronic devices such as flat panel displays, organic light emitting diodes, and transparent electrodes for solar cells^2–4^. The crystal morphology of SnO_2_ can considerably improve the performance of electronic devices, as exemplified by the SnO_2_ nanomaterials that show larger electrical and optical variation than those of bulk SnO_2_^5^.

It is well known that the electrical transport of material plays a central role in the performance of optoelectronic devices. For SnO_2_, studies of its conduction mechanism have provided possible routes for optimising nanocrystalline SnO_2_-based devices^6,7^. Various approaches have been proposed to enhance the electrical conductivity, including changing the grain size^8^ and chemical doping^9^. Applying pressure compression has been shown to be one of the effective approaches to tuning the crystalline structure, the electrical structures and the electrical transport properties of materials, which inspired us to further explore the pressurisation of SnO_2_. A previously reported observation indicated that with application of pressure, SnO_2_ transforms from rutile (tetragonal structure with *P*4_2_*/mnm* symmetry) to a CaCl_2_-type phase (orthorhombic structure with *Pnnm* symmetry) under hydrostatic pressure or an α-PbO_2_-type phase (orthorhombic structure with *Pbcn* symmetry) at ~12 GPa under non-hydrostatic pressure conditions. Both α-PbO_2_-type and CaCl_2_-type phases were found to transform to a modified fluorite-type phase (cubic structure with *Pa*-3 symmetry) at with further compression up to 21 GPa. How compression tunes the structural properties of SnO_2_ has been well studied^10–18^. However, very few works have focused on the effects of pressure on the electrical resistivity of SnO_2_^19^, which significantly precludes our understanding of the conduction mechanism of nanocrystalline SnO_2_.

Since SnO_2_ is a semiconducting dielectric material and widely applied in optoelectronic devices, its resistivity and dielectric properties are two important factors to characterize this material. For example, the resistance and dielectric change will affect the feature and efficiency of device, such as thermal loss, leakage current, refractive index, signal responding speed and so on. SnO_2_ as a transparent conductive electrode material, the resistivity affects power conversion efficiency (PCE) of devices, while the dielectric affects light transmittance. The optimization of device development based on SnO_2_ material requires a better understanding of the dielectric properties^20^.

Herein, we track the evolutions of electrical, structural, and dielectric properties of SnO_2_ nanoparticles *in situ* under compression using a combination of AC impedance spectroscopy and X-ray diffraction (XRD) experiments. The electrical and dielectric properties of nanocrystalline SnO_2_ under compression have been well discussed.

## Methods

Our sample is nanocrystalline SnO_2_ powder (99.996%) purchased from Alfa Aesar Co. High pressure was obtained by a diamond anvil cell (DAC), with one pair of diamonds with a culet of 300 μm in diameter and a T-301 steel gasket. A 150 μm hole with was drilled in the centre of the indentation area. To insulate the metallic gasket and electrodes, the hole was covered with compressed alumina (Al_2_O_3_) and epoxy powders. Another hole with a diameter of 100 μm was then drilled in the centre of the compressed Al_2_O_3_ for use as a sample chamber. Thin-film Mo electrodes were magnetically sputtered onto the diamond anvil, forming a pair of capacitance block-electrodes for electrical transport measurements. More detailed information about the configuration of the parallel plate was reported in previously^21^. AC impedance spectroscopy measurements were conducted on a Solartron 1260 impedance analyser equipped with a Solartron 1296 dielectric interface with a frequency range from 10^−1^ to 10^7^ Hz. During the measurements, the DAC was shielded with a metal box to avoid any electromagnetic background and pseudo linear responses from the inner structure of the cell.

*In situ* high-pressure XRD experiments were conducted at BL15U1 of the Shanghai Synchrotron Radiation Facility (SSRF) and BL4W2 of the Beijing Synchrotron Radiation Facility (BSRF), using angle-dispersive XRD mode (λ = 0.6199 Å). Argon was used as the pressure transmitting medium. The instrument parameters, including the distance between the sample and detector, were calibrated using a CeO_2_ standard material. Ruby chips were loaded into the chamber near the sample to calibrate the pressure.

The first-principles calculations were performed based on the density functional theory and the pseudo potential method on the standard CASTEP program in the Material Studio package. The effects of electron−electron exchange correlation are depicted by CA-PZ functional in Local Density Approximation (LDA). The geometric optimization of the unit cell was conducted with the Broydene−Fletchere−Goldfarbe− Shanno (BFGS) minimization algorithm. Integration in the Brillouin zone was performed by use of special *k* points generated with 7 × 7× 11, 8 × 8 × 11, and 8 × 8 × 8 mesh parameter grids for the tetragonal, the orthorhombic, and the cubic phase, respectively. A plane-wave cutoff energy of 750 eV was set up for the two phases to guarantee the convergence of the enthalpy calculations.

## Results and Discussion

### Impedance analysis

**Figure 1:**
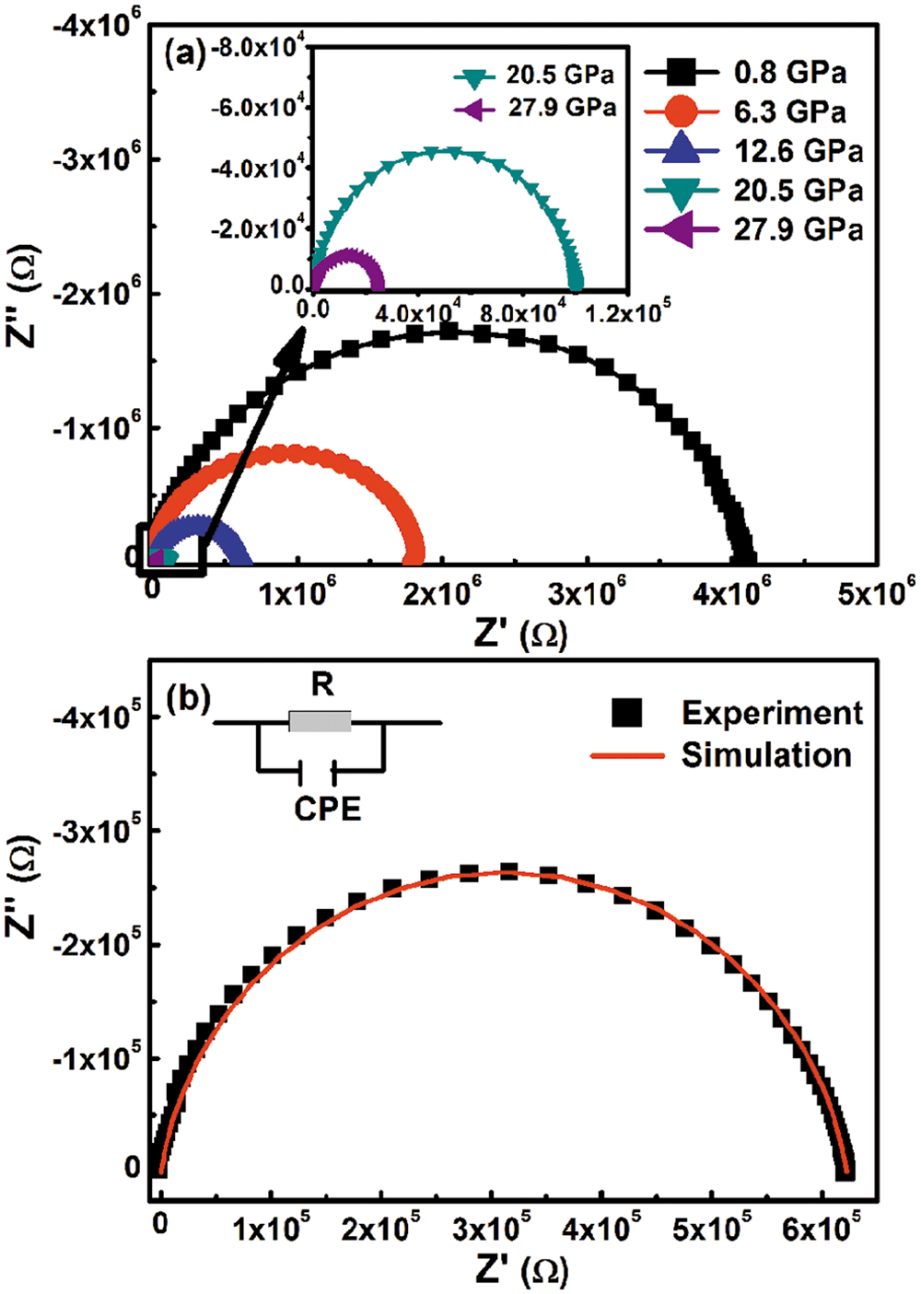
(**a**) Real Z′ verses imaginary Z″ components of impedance, for the SnO_2_ nanoparticles at various compression pressures. The inset shows a magnification of the data at 20.5 and 27.9 GPa. (**b**) Equivalent circuit used to describe the electrical properties of the SnO_2_ nanoparticles.

The average grain size of SnO_2_ nanoparticles is about 50 nm, and it is homogeneous. The scanning electron microscopy (SEM) pattern is shown in Supplementary Fig. 1. The complex impedance spectra of the SnO_2_ nanoparticles at various compression pressures are shown in Fig. 1(a). A single semi-circular response corresponding to grain interiors is observed at every pressure. The components related to the grain boundaries and electrode effects were too weak compared with those of the grain contribution to be distinguished. With application of pressure, the semicircle related to the grain effect collapses considerably but the shape of the arcs remains unchanged.

**Figure 2:**
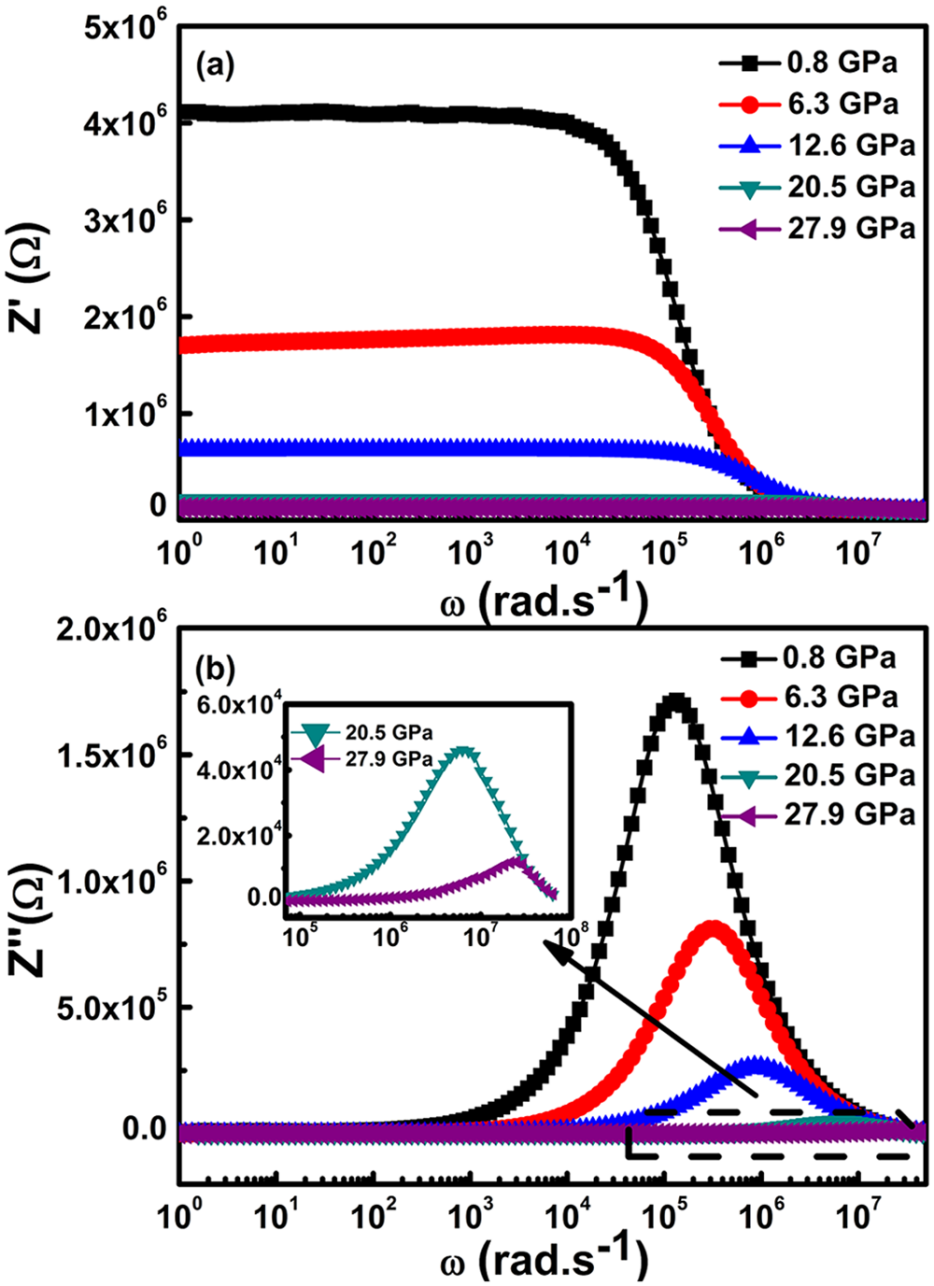
The real *Z*′ (**a**) and imaginary *Z*″ (**b**) components of impedances as a function of the angular frequency, ω, for the SnO_2_ nanoparticles at different compression pressures.

The representations of the real and imaginary parts of the impedance are given as a function of angular frequency. The purpose is to evaluate the relaxation frequency of the most resistive contribution, which is related to the type and strength of the electrical relaxation in the SnO_2_ nanoparticles. The dependences of the experimental values of *Z*′ and *Z*″ on angular frequency *ω* at different pressures are shown in Fig. 2(a) and (b), respectively. In the low frequency region, the amplitude of *Z*′ in Fig. 2(a) is typically higher at lower pressures. It then gradually decreases with increasing frequency and pressure, indicating an increase in the AC conductivity of the SnO_2_ nanoparticles. In Fig. 2(a), the value of *Z*′ decreases with increasing frequency and attains a constant value at high frequencies at all pressures. Similar behaviour has been reported previously, which suggests a possible release of space charge, and a consequent lowering of the barrier properties in the SnO_2_ nanoparticles^22,23^.

Figure 2(b) shows that *Z′′* initially reaches a maximum value (*Z′′*_max_) and then decreases with increasing frequency at all measured pressures. The maximum appears when the hopping frequency of localised electrons is approximately equal to the frequency of the applied electric field. The average position of the maximum regularly shifts to higher frequencies with increasing pressure, which demonstrates the presence of a pressure-dependent electrical relaxation phenomenon^24^. Furthermore, the asymmetric broadening of the maxima with increasing pressure suggests a spread of relaxation time in the SnO_2_ nanoparticles^25^. Finally, all *Z*″ merge in the high-frequency region under all measured pressures because the frequency-dependent relaxation process of space charge becomes fast and hence leads to the space charge polarisation decreasing with increasing frequency^26^.

**Figure 3:**
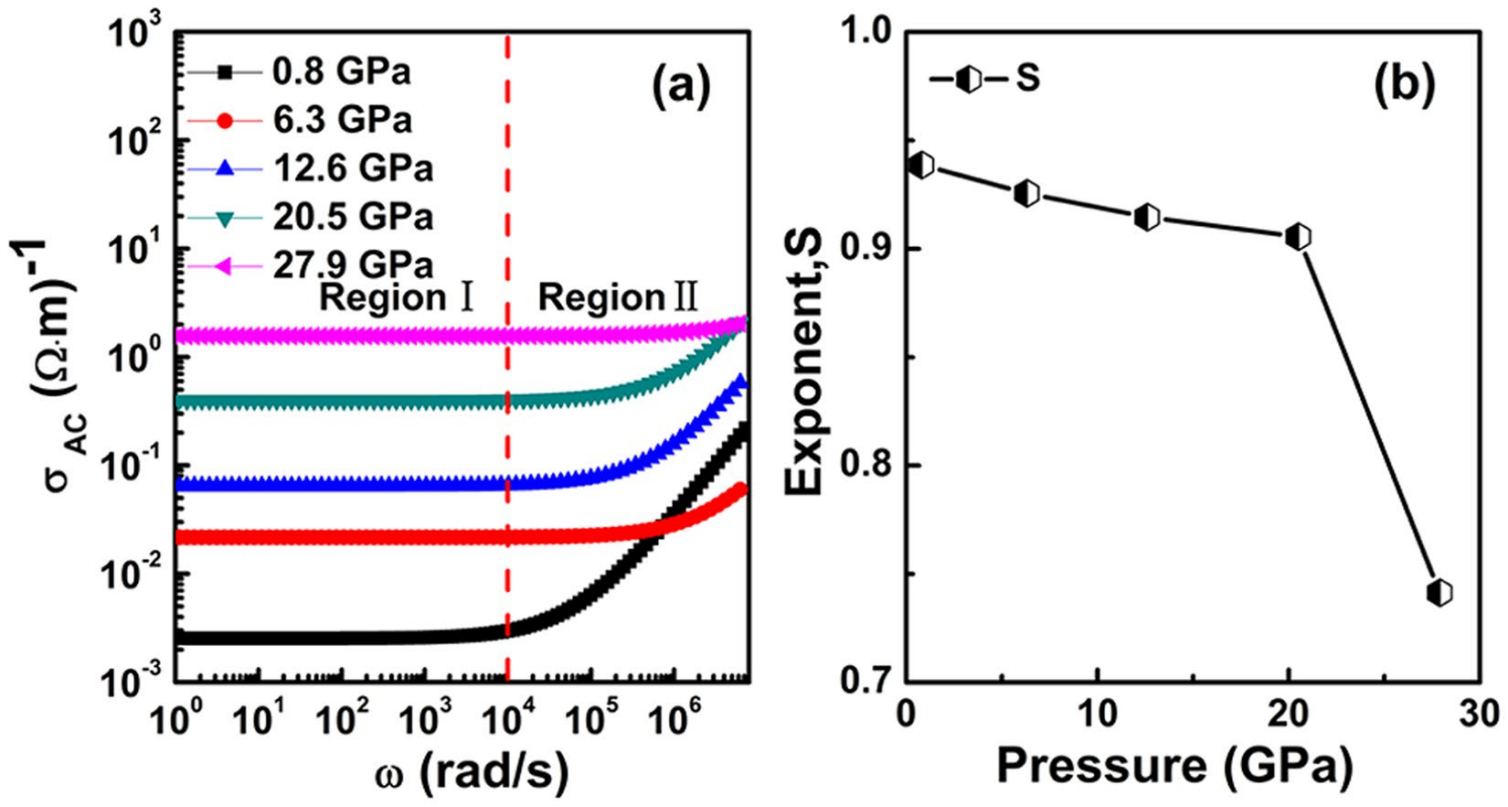
(**a**) The AC conductivity σ_AC_ versus frequency *f* for SnO_2_ nanoparticles at different compression pressures. (**b**) Variation of exponent *S* with pressure.

The AC conductivity *σ*_AC_ of a material is represented by two parts: *σ*(*ω*) = *σ*′(*ω*) + *σ*″(*ω*), in which *σ*′(*ω*) = 2π*ε*_*0*_*ε*″ is the real part, and *σ*″(*ω*) = −2*πε*_*0*_*ε*′ is the imaginary part. The AC conductivity of the SnO_2_ nanoparticles as a function of frequency *f* at different pressures is shown in Fig. 3(a). The conductivity pattern can be divided into two parts. At low frequencies, the AC conductivity is weakly frequency dependent at each pressure which corresponds to DC conductivity. At higher frequencies, the AC conductivity shows dispersion that is characteristic of *ω*^*s*^. The phenomenon of the dispersion of conductivity generally obeys the power law relationship of Jonscher: *σ*_AC_ = *σ*_DC_ + *Aω*^*S*^, where *σ*_DC_ is the DC conductivity, A is a complex proportionality constant and ω is the angular frequency. The exponent *S* is a frequency-dependent parameter of value less than unity, and its dependence on pressure determines the conduction mechanism in the material^27^. The variation of *S* with pressure in Fig. 3(b) is limited to 0.6 < *S* < 1 and *S* decreases with the increase in pressure, which indicates a hopping conduction mechanism in the SnO_2_ nanoparticles^27–29^. The observed dispersion of conductivity with frequency and pressure is in general agreement with the prediction of the correlated barrier hopping (CBH) model^27,30^.

**Figure 4:**
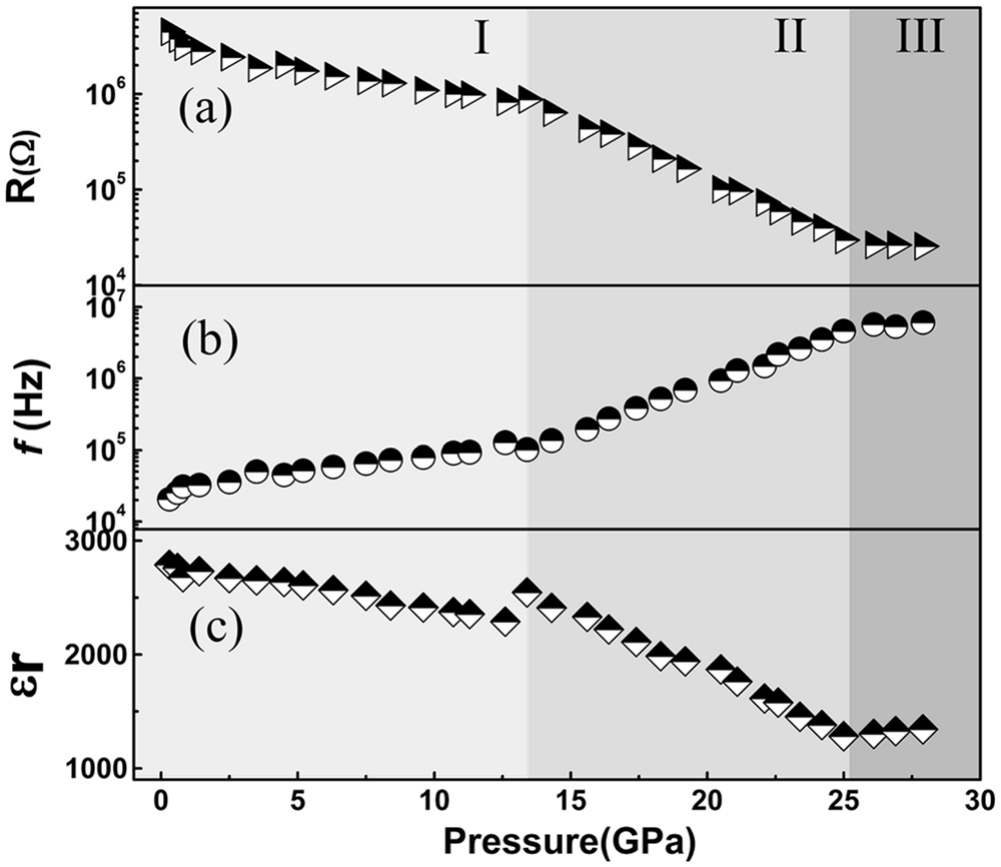
Resistance R, relaxation frequency *f*, and relative permittivity *ε*_r_ of the SnO_2_ nanoparticles at various compression pressures.

To quantify the characteristic relaxation frequencies and electrical resistances of the SnO_2_ nanoparticles, Nyquist plots have been modelled using the Z-view software package. As shown in the insert in Fig. 1(b), an equivalent circuit formed by the parallel combination of the resistance R and constant phase element (CPE) was used for this purpose. Fitting data for the resistance and relaxation frequency as a function of pressure are shown in Fig. 4. With increasing compression pressure, each parameter shows three linear regions with different slopes. The slope changes occur at 13.4 and 25.0 GPa. In fact, most anomalies of the electrical parameters usually coincide with crystal structure transition. To determine the correlation between the electrical transport properties and the structure of SnO_2_ nanoparticles, we performed high pressure synchrotron XRD experiments.

**Figure 5:**
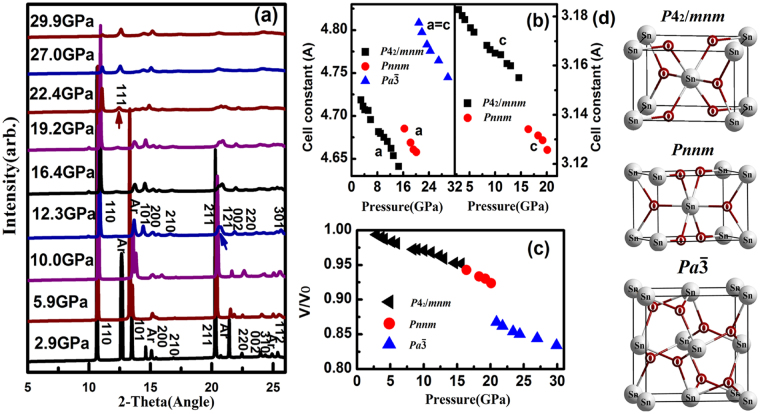
(**a**) XRD patterns of the SnO_2_ nanoparticles at various compression pressures. (**b**) The relative volume of the SnO_2_ nanoparticles as a function of compression pressure. (**c**) Cell constants of the SnO_2_ nanoparticles as a function of compression pressure. (**d**) Crystal structures of the three high-pressure polymorphs of SnO_2_ nanoparticles.

Figure 5(a) shows the collected XRD data at different pressures. From 2.9 to 12.3 GPa, the XRD pattern of nanocrystalline SnO_2_ is consistent with a high-crystallinity rutile structure (*P*4_2_*/mnm)*. At higher pressures, the (211) diffraction peak begins to split and broaden into the (101) and (200) reflections. This suggests that an orthorhombic phase is formed (i.e., a CaCl_2_-type structure with space group *Pnnm*). No discontinuities in the relative volume and cell constant are observed, as shown in Fig. 5(b) and (c). This indicates that the phase transition could be second order. The diffraction pattern at 22.4 GPa shows a weak peak emerging between the strong (110) and (101) peaks of the rutile structure, indicating the onset of a phase transition in the SnO_2_ nanoparticles. As the pressure increases, the intensities of peaks related to the rutile phase decrease, and the intensities of peaks originating from the new phase increase. The new peaks could be assigned to the high-pressure cubic fluorite ($Pa\bar{3}$) phase, because of the typical characteristics of the cubic fluorite phase ($Pa\bar{3}$) in the (111) direction. This conclusion is consistent with an earlier report^31^. The changes in the electrical parameters at 13.4 and 25.0 GPa are therefore attributed to the rutile-to-CaCl_2_ and CaCl_2_-to-fluorite phase transitions, respectively. The rutile, CaCl_2_ and fluorite structures are described in Fig. 5(d). Compared with the synchrotron XRD experiment, the transitions pressures shift approximately 1‒3 GPa towards higher pressure, which can be attributed to the pressure transmitting medium. To avoid the introduction of additional effects, our electrical experiments did not use the pressure medium, but we used argon in the high-pressure synchrotron XRD experiment. Neglecting the pressure transmitting medium can give rise to deviatoric stresses at different pressures. The absence of a pressure-transmitting medium in an electrical experiment can cause deviatoric stress.

**Figure 6:**
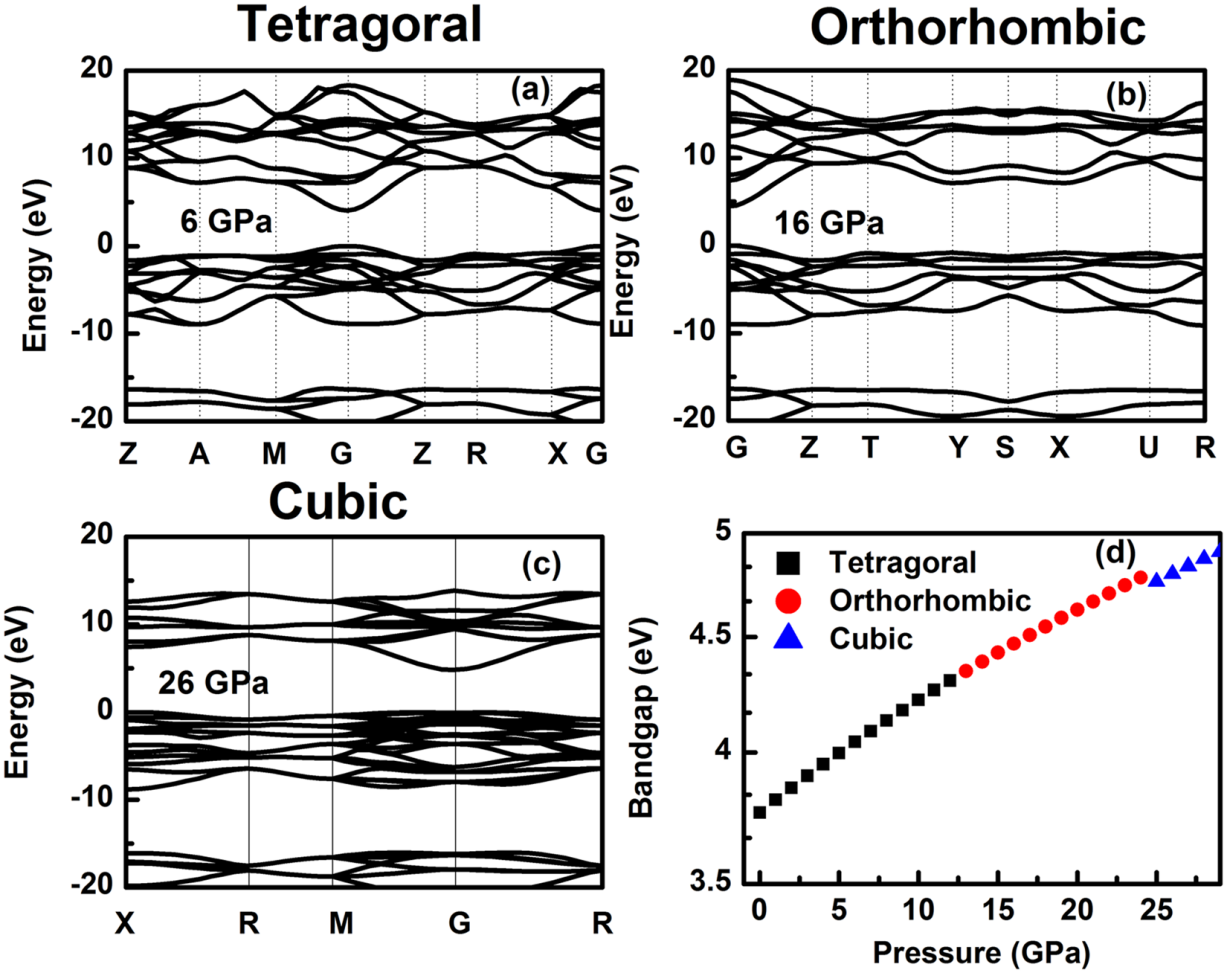
The band structure of (**a**) the tetragonal phase at 6 GPa and (**b**) the orthorhombic phase at 16 GPa, (**c**) the cubic phase at 26 GPa. (**d**) The pressure dependence of energy bandgap in three phases.

In general, the compression always causes an energy gap narrow effect and then results the conductivity increase of the compressed sample. For SnO_2_, the situation is different. We have calculated the band gap of SnO_2_ at different phases (as shown in Fig. 6) and the pressure induced band gap broaden has been found at every phase. This result shows the intrinsic band gap change will result the conductivity decrease at every phase. The effect of band gap change on conductivity is negative. However, our experimental result shows that the conductivity of SnO_2_ increases with pressure increasing.

According to the Arrhenius relationship, the relaxation frequency of the grain can be expressed by the following equation:

1 $$f={f}_{0}exp(-H/{\kappa }_{B}T)$$

where *H* represents the activation energy of the grain, and corresponds to the energy barrier that carriers pass over in the grain interior. *k*_*B*_ is the Boltzmann constant, and *T* is the temperature. Assuming that *f* and *H* are the only functions of pressure, and if *f*_*0*_ remains constant, then:

2 $$d({\rm{l}}{\rm{n}}{\rm{f}})/dP=-(1/{k}_{B}T)(dH/dP)$$

Linear fitting of the curve of ln*f* versus *P* yields the pressure dependence of the activation energy *dH*/*dP*, as shown in Table 1. It is seen that the activation energy decreases with increasing pressure in the tetragonal and orthorhombic phases but slightly decreases in the cubic phase. The decrease in activation energy under pressure indicates that pressure plays an important role in decreasing the energy barriers, which consequently enhances the conductivity. The electrical conduction of semiconducting metal oxides such as SnO_2_ and ZnO strongly depends upon lattice vacancies at oxygen sites^6,19^. The conductivity may also result from the migration of charged particles of SnO_2_, such as *O*^−^, *O*^2−^, and ${O}_{2}^{-}$, or the migration of oxygen defects across the grain interior. A pressure increase leads to an energy barrier decrease in the grain of the SnO_2_ nanoparticles, which results in the resistance decrease shown in Fig. 4(a). Another reason for conductivity growing in SnO_2_ with pressure increasing is due to the connectivity between neighbor grains in compressed SnO_2_ nanoparticles is improved.

**Table 1:** Pressure dependence of activation energy *dH*/*dP* within the grain interior.

Phase	Pressure (GPa)	*dH*/*dP* (meV/GPa)	Error (%)
tetragonal	0.8‒12.6	−2.84	0.228
orthorhombic	13.4‒24.2	−8.45	0.198
cubic	25.0‒27.9	−0.941	2.745

### Dielectric properties

With the parallel-plate electrode model, the relative permittivity (*ɛ*_r_) of the grain as a function of pressure is given by Eq. 3:

3 $${\varepsilon }_{r}(P)=d/(2\pi Rf{\varepsilon }_{0}S)$$

where *d* is the sample thickness, *ɛ*_*0*_ is the vacuum permittivity, *S* is the area of the electrode, and *f* is the relaxation frequency of the grain. The change in *ɛ*_*r*_ with increasing pressure is shown in Fig. 4(c). Two abnormal slope changes occur at 13.4 and 24.2 GPa. The high-pressure XRD experiments indicate that the SnO_2_ nanoparticles are subjected to tetragonal-to-orthorhombic and orthorhombic-to-cubic phase transitions under high pressure. Therefore, the abnormal slope changes in Fig. 4(c) are attributed to these phase transitions. The relative permittivity is measured at a single relaxation frequency and is a static permittivity. The variation of relative permittivity under compression indicates that the dielectric performance of SnO_2_ nanoparticles is modulated by pressure. The pressure makes the polarization rate of SnO_2_ nanoparticles decrease, which is beneficial to its application in the micro-circuit integration.

The complex permittivity is measured in the alternating electric field which known as the dynamic permittivity. The complex dielectric constant *ε*′ and dielectric loss *ε*″ are determined from the following relationships:

4 $$\varepsilon ^{\prime} =Z^{\prime\prime} /[\omega {C}_{0}({Z^{\prime} }^{2}+{Z^{\prime\prime} }^{2})]$$

5 $$\varepsilon ^{\prime\prime} =Z^{\prime} /[\omega {C}_{0}({Z^{\prime} }^{2}+{Z^{\prime\prime} }^{2})]$$

where *C*_0_ is the vacuum capacitance of the cell.

**Figure 7:**
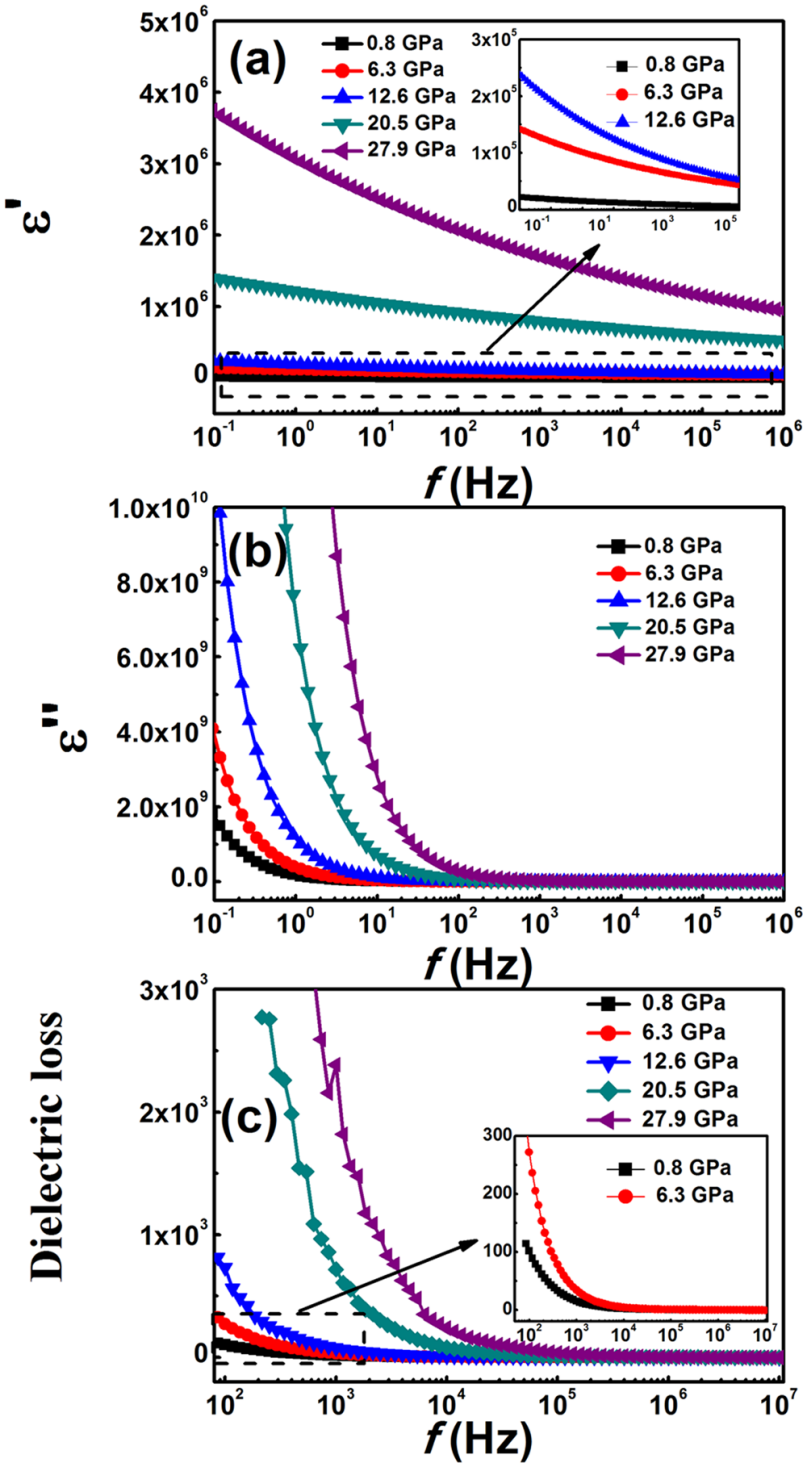
Dependence of the (**a**) dielectric constant *ε*′ and (**b**) dielectric loss *ε*″ on frequency *f*, as a function of compression pressure. (**c**) Variation in the dielectric loss tangent (tanδ) of the SnO_2_ nanoparticles with frequency *f*, for different compression pressures.

Figure 7 shows the frequency dependences of *ε*′ and *ε*″ as functions of pressure. As seen, the dielectric constant *ε*′ of SnO_2_ nanoparticles increases with pressure in all frequency regions. The dielectric behaviour is strongly related to their conduction mechanism^32,33^. At higher pressure, the charge carrier mobility and the rate of hopping increase, hence, the dielectric polarisation increases, causing an increase in the dielectric constant. With increasing frequency, the charge carrier hopping cannot follow the alternating current which leads to a decrease polarisation. This type of polarization mechanism has been discussed by Aziz *et al*.^34^.

*ε*″ is a measure of the dissipated energy in the dielectric material, due to the presence of an applied electric field. The evolution of *ε*″ as a function of frequency at different pressures is shown in Fig. 7(b). At low frequencies, *ε*″ decreases rapidly with increasing frequency and then becomes constant at higher frequencies. The fast decrease in *ε*″ at lower frequencies is attributed to space charge polarisation in the pellet sample. *ε*″ is almost constant at higher frequencies, because of an inability to follow the applied external electric field. This type of polarisation can be explained using the Maxwell-Wagner-Sillars theory of dielectric dispersions^35,36^. The *ε*″ peak position shifts towards higher frequency with increasing pressure, showing that the relaxation process increases with the pressure. This could be because, as the pressure increases, the polar charge carriers are freer to orient, allowing them to adjust to the changing electric field.

Additional information that can be deduced from the loss tangent tanδ (=*ε*″/*ε*′), which is proportional to the amount of energy dissipated in a dielectric material. The variation of tanδ with frequency at different pressure is shown in Fig. 7(c). It can be seen that tanδ decreases rapidly at low frequencies, and becomes almost constant at high frequencies. This confirms that the Maxwell-Wagner relationship is responsible for the enhanced dielectric permittivity at low frequency. The decrease in tanδ at low frequencies suggests that the present SnO_2_ nanoparticles could be used in high frequency devices.

### Electric modulus analysis

Investigating the form of the complex electric modulus *M* is another approach to exploring the electrical properties of the SnO_2_ nanoparticles. This approach can also magnify other effects present in the sample because of different relaxation time constants. The real (*M*′) and imaginary (*M*″) parts of the complex electric modulus were obtained from the impedance data in a conventional way according to the following equations^37^:

6 $$M^{\prime} =\varepsilon {C}_{0}Z^{\prime\prime} $$

7 $$M^{\prime\prime} =\varepsilon {C}_{0}Z^{\prime} $$

**Figure 8:**
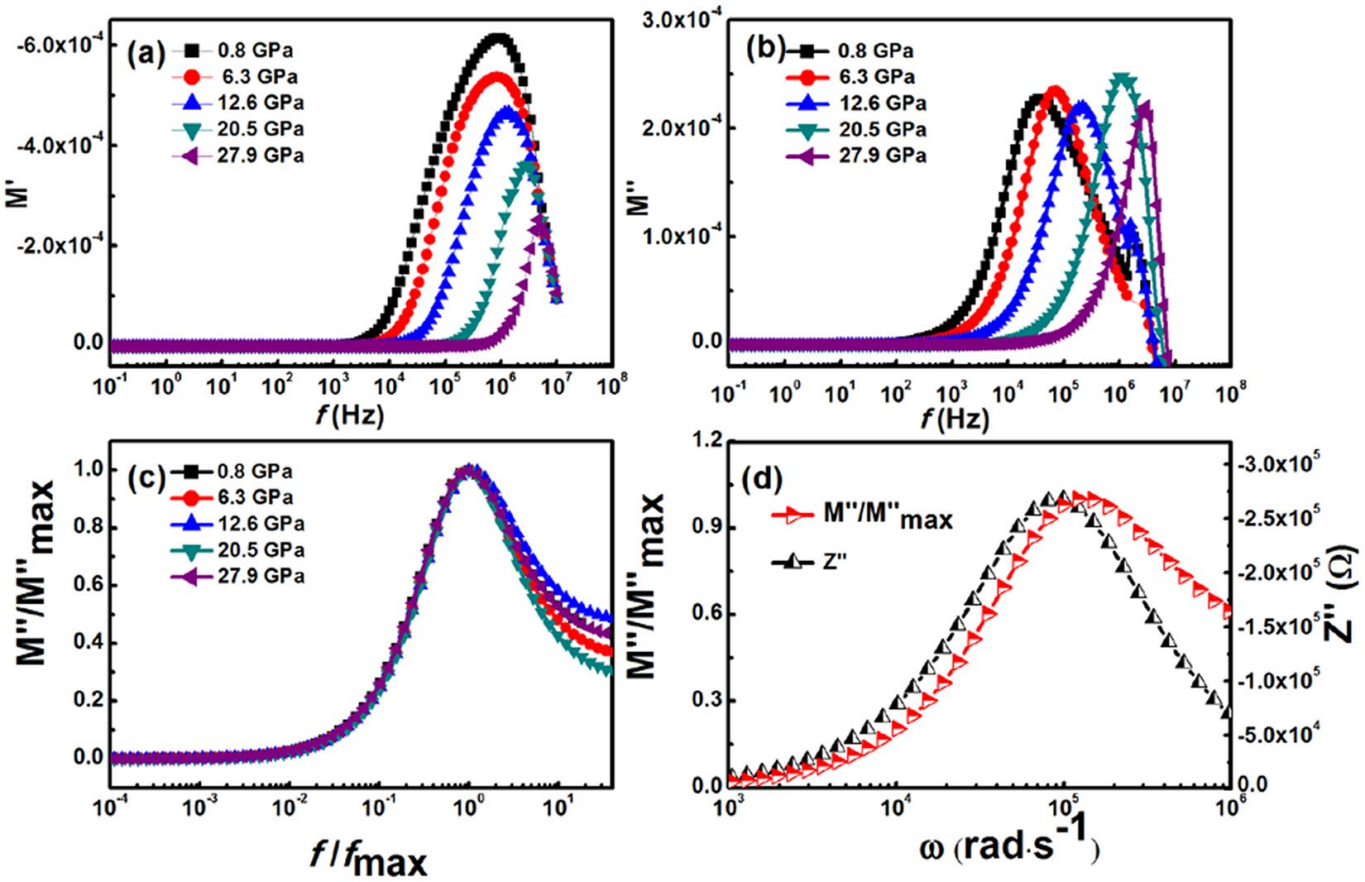
(**a**) Dependence of the (**a**) real *M*′ and (**b**) imaginary *M*″ parts of the complex modulus on frequency for the SnO_2_ nanoparticles at different compression pressures. (**c**) Normalised imaginary part of the electric modulus *M*″/*M*″_max_ versus normalised frequency *f*/*f*_max_ for the SnO_2_ nanoparticles under different compression pressures. (**d**) *M*″/*M*″_max_ and *Z*″ versus angular frequency *ω*, for the SnO_2_ nanoparticles at a compression pressure of 12.6 GPa.

*M*′ and *M*″ for our SnO_2_ nanoparticles at various compression pressures are shown in Fig. 8(a) and (b), respectively. In Fig. 8(a), *M*′ tends to zero at low frequencies, suggesting that the interface effect tends to be eliminated in the modulus representation. At high frequencies, *M*′ displays a maximum value corresponding to *M*′_max_, which can be attributed to the phenomenon of conduction due to the mobility of charge carriers at a small distance. The plots in Fig. 8(b) are characterized by the presence of a relaxation peak. At lower frequencies, charge carriers can move freely over longer distances, up to a certain frequency (peak maximum). A further increase in frequency confines carriers to potential wells. Therefore, the region where the peak occurs indicates the transition from long-range to short-range mobility with increasing frequency^38^.

Figure 8(c) shows the dielectric spectra of the normalised imaginary part of the electric modulus *M*″/*M*″_max_ as a function of the normalised frequency *f*/*f*_max_, at different pressure. The data under different pressures overlap almost perfectly, except for their high-frequency tails. This indicates that all dynamic processes occurring on different time scales exhibit a distribution of relaxation times that is independent of compression pressure.

The combined plot of *M*″/*M*″_max_ and Z″ versus angular frequency can distinguish whether the short- or long- range movement of charge carriers dominates the relaxation process. Figure 8(d) shows plots of *M*″/*M*″_max_ and *Z*″ versus angular frequency, at a compression pressure of 12.6 GPa. The peak maxima of the two curves do not occur at the same frequency. This indicates that the relaxation process is dominated by the short range movement of charge carriers, and thus departs from ideal Debye-type behaviour^39^.

## Conclusions

The electric and dielectric properties of SnO_2_ nanoparticles were investigated as a function of frequency and pressure. The pressure-induced structural phase transitions in the SnO_2_ nanoparticles correspondingly change their electrical transport and dielectric properties. Complex impedance plots reveal only one semi-circular curve, which is attributed to the grain effect. The AC conductivity and electric modulus studies suggest hopping-type conduction in this system. Frequency-dependent AC conductivity data obey the universal power law at each compression pressure. The dielectric constant and dielectric loss factor decrease with increasing frequency and pressure, which can be interpreted by the Maxwell-Wagner-Sillars model. Furthermore, detailed studies of modulus spectra suggest that the SnO_2_ nanoparticles exhibit a non-Debye type of relaxation mechanism. These findings hint at the potential of SnO_2_ nanoparticles in dielectric applications.

## Electronic supplementary material

Supplementary Information
