# Supplementary material for: Effects of high pressure on the electrical resistivity and dielectric properties of nanocrystalline SnO2
Source: Sci Rep. 2018 Mar 23;8:5086. doi: 10.1038/s41598-018-22965-8 (PMC5865191; doi:10.1038/s41598-018-22965-8)
Supplement: Supplementary file 1 — Supplementary Information [file 41598_2018_22965_MOESM1_ESM.doc]

### Supplementary Information

**Effects of high pressure on the electrical resistivity and dielectric properties of nanocrystalline SnO_2_**

Wenshu Shen^1^, Tianji Ou^1^, Jia Wang^1^, Tianru Qin^1^, Guozhao Zhang^1^, Xin Zhang^1^，Yonghao Han^1^, Yanzhang Ma^2,3^, Chunxiao Gao^1^*

*SI correspondence to:

Chunxiao Gao, Email: cc060109@qq.com

**Supplementary Fig. 1. SEM microphotograph of nanocrystalline SnO_2_.** The average particle size of the sample, measured by scanning electron microscope (SEM) as shown in Fig S1, was about 50 nm.

**
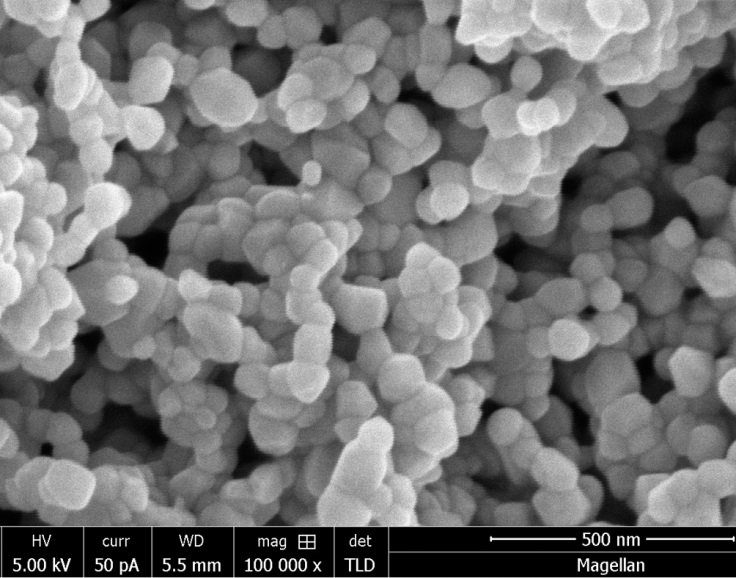
**
